# Validation of an information–motivation–behavioral skills model of self-care among Chinese adults with type 2 diabetes

**DOI:** 10.1186/1471-2458-13-100

**Published:** 2013-02-04

**Authors:** Junling Gao, Jingli Wang, Yaocheng Zhu, Jinming Yu

**Affiliations:** 1 School of Public Health, Fudan University; Key Laboratory of Public Health Safety, Ministry of Education, Shanghai, 200032, China; 2 Dachang Center of Primary Health Care, Shanghai, 200442, China

**Keywords:** Type 2 diabetes, Self-care, Self-efficacy, Social support, Lipid ratios

## Abstract

**Background:**

Self-care is a crucial component of diabetes management. But comprehensive behavior change frameworks are needed to provide guidance for the design, implementation, and evaluation of diabetes self-care programs in diverse populations. We tested the Information–Motivation–Behavioral Skills (IMB) model in a sample of Chinese adults with Type 2 diabetes.

**Methods:**

A cross-sectional study of 222 Chinese adults with type 2 diabetes was conducted in a primary care center. We collected information on demographics, provider-patient communication (knowledge), social support (motivation), self-efficacy (behavioral skills), and diabetes self-care (behavior). The values of total cholesterol (TC), low-density lipoprotein cholesterol (LDL-C) and high-density lipoprotein cholesterol (HDL-C) were also obtained. Measured variable path analyses were used to the IMB framework.

**Results:**

Provider-patient communication (β = 0.12, p = .037), and social support (β = 0.19, p = .007) and self-efficacy (β = 0.41, p < .001) were independent, direct predictors of diabetes self-care behavior. Diabetes self-care behaviors had a direct effect on TC/HDL-C (β = −0.31, p < .001) and LDL-C/HDL-C (β = −0.30, p < .001).

**Conclusions:**

Consistent with the IMB model, having better provider-patient communication, having social support, and having higher self-efficacy was associated with performing diabetes self-care behaviors; and these behaviors were directly linked to lipid control. The findings indicate that diabetes education programs should including strategies enhancing patients’ knowledge, motivation and behavioral skills to effect behavior change.

## Background

The world prevalence of diabetes among adults (aged 20–79 years) was 6.4% in 2010, and will increase to 7.7% by 2030. Between 2010 and 2030 [1], estimates predict a 69% increase in adults with diabetes in developing countries and a 20% increase in developed countries. The prevalence of diabetes is high in China and continues to increase. Overall, 92.4 million Chinese adults 20 years of age or older (9.7% of the adult population) have diabetes, and in 60.7% of these cases, the diabetes is undiagnosed [2]. Cardiovascular disease (CVD) is the major cause of morbidity and mortality for individuals with diabetes and the largest contributor to the direct and indirect costs of diabetes [3]. Patients with type 2 diabetes (T2DM) have an increased prevalence of lipid abnormalities, which may cause CVD. It is reported that most patients with T2DM could have lipid abnormalities at varying degrees, characterized by increased levels of total cholesterol (TC) and low-density lipoprotein cholesterol (LDL-C) and decreased high-density lipoprotein cholesterol (HDL-C). At recent years, more data support that the lipid ratios, including TC/HDL-C and LDL-C/HDL-C are more sensitive in reflecting the morbidity and severity of CVD than individual lipid levels [4-7].

Self-care behaviors influence glycemic control [8-10] and lipid levels [11,12]. Typically, these behaviors include monitoring for signs/symptoms of the disease, managing diet, exercising, testing blood glucose, taking medications, inspecting feet for early indications of compromised circulation, stopping smoking, and controlling alcohol consumption [13,14]. While self-care is crucial in diabetes management, few patients engage in the full set of self-care behaviors at recommended levels [15,16]. Because most patients with chronic diseases, including diabetes, receive health care at primary care centers, it is important that primary care providers understand how to encourage self-care behaviors and improve health outcomes.

Many models have informed diabetes educational efforts [17]. However, no single conceptual framework to date was comprehensive enough to link attributes of high quality diabetes care to self-care processes and diabetes outcomes [18]. So some researchers [19,20] applied a comprehensive, theoretical model of health behavior change, known as the Information–Motivation–Behavioral Skills (IMB) model [21,22] to explain diabetes self-care and its effects on glycemic control. To our knowledge, no study examined the utility of the IMB model in self-care among Chinese adults with T2DM.

The IMB model identifies three core determinants of the initiation and maintenance of health behaviors: accurate *information* that can be readily translated into health behavior performance; personal and social *motivation* to act on such information; and *behavioral skills* to confidently and effectively implement the health behavior [22]. *Information* refers to funds of behavior-relevant accurate information and faulty heuristics or mis-information about a health behavior. We used provider-patient communication (PPC) as indicative of information. Because a major purpose of provider-patient communication is to exchange information about the disease and its treatments, so a positive communication style may improve patients’ understanding and recall of information about diseases [23]. Furthermore better provider-patient communication was positively associated with better information and self-efficacy in Chinese people with T2DM [24]. *Motivation* is comprised of two components, personal and social motivation. Personal motivation is a function of one’s beliefs about the consequences of a behavior and evaluations of these consequences. Social motivation involves perceiving normative support for a health behavior and being motivated to comply with these referent others’ wishes. *Behavioral skills* include objective and perceived skills for performing the behavior and a sense of self-efficacy for doing so. Social support and self-efficacy served as the measures of social motivation and behavioral skill in current study.

**Figure 1:**
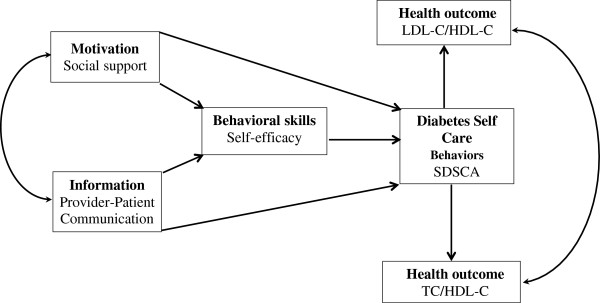
Hypothesized Information–Motivation–Behavioral skills model of diabetes self-care.

The purpose of the current study is to test whether the IMB model can explain the self-care behaviors of Chinese diabetes patients. Based on the theoretical underpinnings of the IMB model, we hypothesized that PPC, and social support would affect diabetes self-care behavior directly and indirectly through self-efficacy. Only self-care behavior was predicted to relate to lipid ratios in Chinese adults with T2DM (Figure 1).

## Methods

A cross-sectional, observational study was conducted at a primary care center in Shanghai, China between June and October 2011. Participants were eligible if they received their usual care at the primary care center and had a diagnosis of T2DM. We excluded participants if they could not complete the survey because of physical or cognitive impairments. All participants provided written informed consent. The Institutional Review Board of the School of Public Health, Fudan University, approved the study.

### Data and procedure

Research assistants reviewed the electronic clinic roster to identify eligible participants. Two hundred twenty-two participants of 274 eligible participants (response rate = 81%) were consented and completed the study. The consented eligible participants were invited to the center to complete the study survey, a physical examination and fasting blood glucose tests. Blood tests, including TC, HDL-C and LDL-C were provided for free as an incentive for participation. We collected data on self-reported age, gender, marital status, education, household income, family history of diabetes, time diagnosed with diabetes and health insurance. Research assistants checked all selfv-reported questionnaires for completeness. Participants’ height, weight, waistline and hipline were measured guided by Chinese guideline on diabetes care [25].

Additional measures included validated surveys of self-efficacy, social support, PPC, and diabetes self-care behavior. Self-efficacy was assessed with the Chinese version of the Diabetes Management Self-Efficacy Scale (C-DMSES) containing 20 items [26]. It assesses the extent to which participants are confident they can manage nutrition, blood sugar monitoring, foot exams, physical exercise and weight, and medical treatment. Participants rated themselves on an 11 point scale ranging from “0 = can’t do at all” to “10 = certain can do”. The mean scores of the 20 items ranging from 0 to 10 were used to assess participants’ self-efficacy.

Self-care behavior was assessed with the 11-item revised Summary of Diabetes Self-Care Activities (SDSCA) scale [27]. Previous studies [26,28] indicated the revised SDSCA was suitable to measure self-care behavior of Chinese diabetes (Cronbach alpha = .70). The RSDSCA measures frequency of self-care activity in the last 7 days for five aspects of the diabetes regimen: general diet (followed healthful diet), specific diet (ate fruits/low fat diet), foot care, blood–glucose testing, exercise and taking recommended diabetes medication. Participants rated themselves form 0 to 7 on each item. The mean scores of the 11 items ranging from 0 to 7 were used to assess participants’ self-care behavior. The Cronbach alpha coefficient of the Chinese version of the revised SDSCA in this study was 0.81, indicating good internal consistency.

Social support and PPC were measured using the Chinese version scales [29] based on The Health Education Impact Questionnaire [30]. Social support scale aims to capture the positive impact of social engagement and support that evolves through interaction with others and the impact may arise from interaction with others sharing similar health-related life experiences, which consists 5 items with a scoring form 0 to 6 where 0 = “strongly disagree” and 6 = “strongly agree”. The mean scores of the 5 items ranging from 0 to 6 were used to assess participants’ social support. High scores indicate high levels of social interaction, high sense of support, seeking support from others. The Cronbach alpha coefficients of social support scale was 0.930. PPC scale covers an individual’s understanding of and ability to interact with a range of health organizations and health professionals. It also measures the confidence and ability to communicate and negotiate with health care providers to get needs met, which consists of 5 items with a scoring range from 0 to 6 where 0 = “strongly disagree” and 6 = “strongly agree”. High scores characterize a person who is confident in their ability to communicate with healthcare professionals and has good understanding of ways to access healthcare in order to get their needs met. The Cronbach alpha coefficients was 0.929.

We calculated lipid ratios (TC/HDL-C and LDL-C/HDH-C) using the results of tests on blood drawn during the physical examination.

### Analysis

Descriptive statistics were performed using SPSS 17.0. Categorical variables were expressed as percentages and continuous variables were expressed as mean ± SD. Measured variable path analysis (MVPA), a form of structural equation modeling, was used to test the relationships among self-efficacy, social support and PPC, and their effect on self-care, and lipid ratios using AMOS 17.0. Simulation research has shown that with a good model and multivariate normal data a reasonable sample size is 200 cases [31]. The parameter estimation method was maximum likelihood. The likelihood ratio χ^2^ tests are reported, but model fit was primarily evaluated with the comparative fit index (CFI), standardized root mean residual (SRMR) and root mean square error of approximation (RMSEA) [32]. All of them test how well an estimated model fits the data structure. A non-significant likelihood ratio χ^2^ test suggests that the data fit the model well, while CFI values exceeding 0.90, SRMR and RMSEA values less than 0.08 indicate adequate model fit [33].

## Results

### Characteristics of participants

Overall, 137 participants were female (61.7%), and most were married (92.3%). Participants were, on average, 54.5 years old (SD = 6.4, range: 44–80), but most were more than 60 years old (78.4%). Fifty-two graduated from technical school or college (23.4%). On average, duration of diabetes was 8.3 years (SD = 6.4, median = 7.0, range: 1–42). Eighty-six participants had a family history of diabetes (38.7%) (see Table 1). The mean value of TC/HDL-C and LDL-C/HDL-C were 3.8 (SD = 1.1, range: 1.0-8.8) and 2.2 (SD = 0.8, range: 0.4-5.5) respectively. Descriptive information on each measure is presented in Table 2.

**Table 1:** Demographic Characteristics of Participants

**Characteristic**	**mean ± SD, or N(%)**
**Gender**	
Male	85 (38.3)
Female	137(61.7)
**Marital status**	
Married	205 (92.3)
Not married	17 (7.7)
**Mean age (years)**	64.5 ± 6.4
**Age categories**	
<60	48 (21.6)
60-69	119 (53.6)
≥70	55 (24.8)
**Education**	
Illiteracy or elementary school	21 (9.5)
Junior high school	85 (38.3)
Senior high school	64 (28.8)
Technical school or college	52 (23.4)
**Family per capita month income** (RMB)	
<2000	102 (45.9)
≥2000	120 (54.1)
**Family history of diabetes**	
Yes	86 (38.7)
No	136 (61.3)
**Clinical symptoms**	
Yes	96 (43.2)
No	126 (56.8)
**Complications**	
Yes	155 (69.8)
No	67 (30.2)
**Duration of diabetes (years)**	
<4	64 (28.8)
5-9	72 (32.4)
10-14	56 (25.2)
≥15	30 (13.5)
**Mean BMI (kg/m**^**2**^**)**	24.9 ± 3.4
BMI categories*	
<24	91 (41.0)
≥24	131 (59.0)

RMB = Ren Min Bi(Yuan).

* BMI ≥ 24 = overweight/obesity suggested by Chinese Diabetes Society(CDS).

**Table 2:** Self-efficacy, Social support, PPC, SDSCA, TC/HDL-C and LDL-C/HDL-C

**Measures**	**mean ± SD**
Self-efficacy	6.9 ± 1.5
Social Support	4.2 ± 0.7
PPC	4.4 ± 1.0
SDSCA	3.4 ± 1.3
TC/HDL-C	3.8 ± 1.1
LDL-C/HDL-C	2.2 ± 0.8

*PPC* Provider-patient communication.

*SDSCA* Summary of Diabetes Self-Care Activities.

### Validation of the IMB model

**Figure 2:**
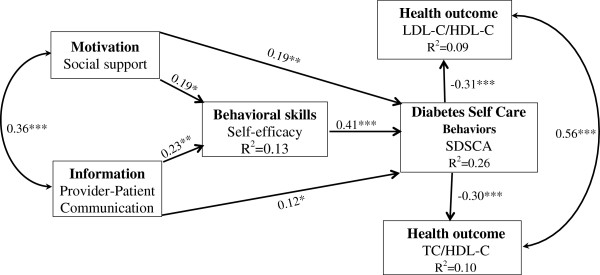
**Evaluated Information–Motivation–Behavioral skills model of diabetes self-care.** Note: Coefficients are standardized path coefficients. Overall model fit, χ^2^ (6, N = 222) =10.74, p = 0.097, CFI = 0.99, SRMR = 0.06, RMSEA = 0.06 (90% CI: 0.00-0.08). For tests of significance of individual paths, *p < .05, **p < .01 and ***p < .001.

The estimated MVPA with parameters and statistical significance of individual paths is shown in Figure 2. The estimated model demonstrated good data fit, χ^2^ (6, N = 222) = 10.74, p = 0.097, CFI = 0.99, SRMR = 0.06, RMSEA =0.06 (90% CI: 0.00-0.09). As indicated in Figure 2, TC/HDL-C and LDL-C/HDL-C were positively associated with each other (r = 0.56, p < .001); there were significant negative direct effects from diabetes self-care behaviors to TC/HDL-C (β = −0.31, p < .001) and LDL-C/HDL_C (β = −0.30, p < .001), explaining 9% of the variability in TC/HDL-C and 10% of the variability in LDL-C/HDL-C. There were significant positive direct paths from self-efficacy (β = 0.41, p < .001), social support (β = 0.19, p = .007) and PPC (β = 0.12, p = .037) to diabetes self-care behaviors, explaining 26% of the variability in the diabetes self-care behaviors. Although social support and PPC had no direct effect on self-care behaviors, both of them had an indirect effect on self-care behaviors (β = 0.08, p = .008; β = 0.09, p = 0.002 respectively) through self-efficacy.

## Discussion

The Information–Motivation–Behavioral Skills (IMB) model of health behavior change [21,22] was used to conceptualize the determinants of diabetes self-care behavior and lipid ratios in a sample of Chinese adults with T2DM. The findings indicated that, overall, the IMB model-based framework for understanding diabetes self-care behaviors was well positioned to explain the sample data. Information and motivation were positively associated with behavioral skill, which was in turn positively associated with diabetes self-care behaviors, which is consistent with previous study [20]. The findings are consistent with the IMB model that proposes that for complex behaviors; information and motivation are insufficient determinants of behavior [34]. While information and motivation may be important in acquiring skills and confidence, even the most informed and highly motivated individuals are likely to struggle with adopting a complex health behavior in the absence of solid practical skills to do so. In other chronic disease contexts, Behavioral skills have mediated the relationship between information and behavior, and motivation behavior [34]. The current study also confirms the previous study findings that information and motivation were also associated with diabetes self-care behaviors directly [19].

Patients with T2DM have an increased prevalence of lipid abnormalities, which may cause CVD. Glycemic control is the primary goal of self-care in T2DM [3], but lipid control is also important to the management of T2DM [35]. Previous studies [19,20,36,37] have well demonstrated diabetes self-care behaviors were associated with better glycemic control. So the current study explored the relationship between diabetes self-care behaviors and lipid ratios because lipid ratios are more sensitive in reflecting the morbidity and severity of CVD than individual lipid level[4-7].the finding indicated that diabetes self-care behaviors were negatively associated with TC/HDL-C and LDL-C/HDL-C. This finding confirms previous studies’ conclusions that self-care behaviors were associated with lipid levels [11,12,38]. The findings of the current study support the utility of the IMB model in organizing core determinants of diabetes self-care behaviors and suggest directions for IMB model-based interventions, where information, motivation, and behavioral skills would be formally targeted through behavioral intervention strategies.

There are limitations to this study that should be acknowledged. First, we were unable to explore the role of moderators (e.g., literacy level, gender) in the evaluated models due to a restricted sample size. Our results speak most clearly to the population under study, but most were older. Therefore, this study should be replicated in different patient groups with larger samples. Secondly, self-report measures were used; objective, observable levels of diabetes self-care behaviors were not assessed. Future research should incorporate ratings of actual performance of diabetes self-care behaviors, such as exercise and taking medication. Thirdly, PPC was used as indicator of information. While better PPC is positively associated with better information and self-efficacy in Chinese people with T2DM [24], future research should investigate the utility of the IMB model using information/knowledge. Fourthly, just social support was measured in the current study; the absence of a measure of personal support is another limitation. In addition, the current study measured these constructs cross-sectionally, and thus can most appropriately speak to associations between constructs observed at a single point in time, not causality. Future research should be conducted to investigate the longitudinal effects of information, motivation and behavior skills on changes in diabetes self-care behaviors.

## Conclusions

Despite these limitations, this study is the first to our knowledge to show the utility of the IMB model in a Chinese sample with T2DM. This study shows that consistent with the IMB model, information, motivation, and behaviors skills are important critical prerequisites to performing self-care behaviors in diabetes. Specifically, having better PPC (information), having social support (social motivation), and having higher self-efficacy was associated with performing diabetes self-care behaviors; and these behaviors were directly linked to lipid control. Chinese patients usually rely on the physician’s suggestions for disease treatment, and receive health care services from primary health care providers. So providers and educators need to enhance patients’ ability of PPC, social support, and behavioral skills in their daily admissions, or tailor educational programs including strategies enhancing patients’ knowledge, motivation and behavioral skills. Such interventions are likely to be more effective at producing behavior change than ad-hoc, knowledge-based programs alone.

## Competing interests

The authors declare no competing interests.

## Authors’ contributions

JG and JY wrote manuscript, analyzed data. JW and YZ researched data, reviewed/edited manuscript and contributed to discussion. All authors read and approved the final manuscript.

## Pre-publication history

The pre-publication history for this paper can be accessed here:

http://www.biomedcentral.com/1471-2458/13/100/prepub
